# Deceased organ donation activity and efficiency in Switzerland between 2008 and 2017: achievements and future challenges

**DOI:** 10.1186/s12913-018-3691-8

**Published:** 2018-11-20

**Authors:** Julius Weiss, Andreas Elmer, Markus Béchir, Christian Brunner, Philippe Eckert, Susann Endermann, Renato Lenherr, Mathias Nebiker, Kai Tisljar, Christoph Haberthür, Franz F. Immer, on behalf of the Comité National du Don d’Organes (CNDO)

**Affiliations:** 1 Swisstransplant, the Swiss National Foundation for Organ Donation and Transplantation, Bern, Switzerland; 2 Zentrum für Innere Medizin, Hirslanden Klinik Aarau, Aarau, Switzerland; 3 Zentrum für Intensivmedizin, Luzerner Kantonsspital, Luzern, Switzerland; 4 Service de Médecine Intensive Adulte et Centre des Brûlés, Centre Hospitalier Universitaire Vaudois (CHUV), Lausanne, Switzerland; 5 Klinik für Anästhesiologie, Intensiv-, Rettungs- und Schmerzmedizin, Kantonsspital St. Gallen, St. Gallen, Switzerland; 6 Chirurgische Intensivmedizin USZ, Universitätsspital Zürich, Zürich, Switzerland; 7 Transplantationszentrum, Direktion Medizin und Universitätsklinik für Intensivmedizin, Inselspital, Universitätsspital Bern, Bern, Switzerland; 8 Medizinische Intensivstation, Universitätsspital Basel, Basel, Switzerland; 9 Klinik Hirslanden, Institut für Anästhesiologie und Intensivmedizin, Zürich, Switzerland

**Keywords:** Organ donation, Transplantation, Donation after brain death (DBD), Donation after cardiocirculatory death (DCD), Donor conversion index (DCI), Public health, Switzerland

## Abstract

**Background:**

Various actions have been taken during the last decade to increase the number of organs from deceased donors available for transplantation in Switzerland. This study provides an overview on key figures of the Swiss deceased organ donation and transplant activity between 2008 and 2017. In addition, it puts the evolution of the Swiss donation program’s efficiency in relation to the situation in the neighboring countries.

**Methods:**

This study is an analysis of prospective registry data, covering the period from 1 January 2008 to 31 December 2017. It includes all actual deceased organ donors (ADD) in Switzerland. Donor data were extracted from the Swiss Organ Allocation System. The “donor conversion index” (DCI) methodology and data was used for the comparison of donation program efficiency in Switzerland, Germany, Austria, Italy and France.

**Results:**

During the study period there were 1116 ADD in Switzerland. The number of ADD per year increased from 91 in 2008 to 145 in 2017 (+ 59%). The reintroduction of the donation after cardiocirculatory death (DCD) program in 2011 resulted in the growth of annual percentages of DCD donors, reaching a maximum of 27% in 2017. The total number of organs transplanted from ADD was 3763 (3.4 ± 1.5 transplants per donor on average). Of these, 48% were kidneys (*n* = 1814), 24% livers (*n* = 903), 12% lungs (*n* = 445), 9% hearts (*n* = 352) and 7% pancreata or pancreatic islets (*n* = 249). The donation program efficiency assessment showed an increase of the Swiss DCI from 1.6% in 2008 to 2.7% in 2017 (+ 69%). The most prominent efficiency growth was observed between 2012 and 2017. Even though Swiss donation efficiency increased during the study period, it remained below the DCI of the French and Austrian donation programs.

**Conclusion:**

Swiss donation activity and efficiency grew during the last decade. The increased donation efficiency suggests that measures implemented so far were effective. The lower efficiency of the Swiss donation program, compared to the French and Austrian programs, may likely be explained by the lower consent rate in Switzerland. This issue should be addressed in order to achieve the goal of more organs available for transplantation.

## Background

Various actions have been taken during the last decade to improve the situation of deceased organ donation in Switzerland. Swisstransplant, the Swiss National Foundation for Organ Donation and Transplantation, and its Comité National du Don d’Organes (CNDO) have implemented a broad range of measures to increase the number of organs available for transplantation in Switzerland. In late 2011, the donation after cardiocirculatory death (DCD) program was reintroduced following an initiative by Swisstransplant [1–3]. In addition, a national monitoring program of all patients who die in intensive care units was developed and introduced by Swisstransplant and the CNDO in 2011, and subsequently extended to patients who die in accident and emergency departments [4, 5]. Since then, this prospective cohort study, called Swiss Monitoring of Potential Donors (SwissPOD), assures that patients who may qualify for organ and tissue donation are detected, and that the next of kin are approached about organ donation in compliance with the Swiss Transplantation Law [4–9].

In the framework of the national action plan “More Organs for Transplantation”, launched in 2013 by the Federal Office of Public Health (FOPH) and the cantons, Swisstransplant and the CNDO were commissioned to develop and implement further measures to advance deceased organ donation in Switzerland [10]. These measures included the training of medical staff, analyzing and optimizing processes and improving quality management and establishing appropriate structures and resources [10–12]. The action taken so far resulted in a notable increase of deceased organ donation since 2012 [13]. However, it seems that the federal action plan’s main goal, a donation rate of at least 20 donors after brain death (DBD) per million of population in 2018, may not be achieved [10]. This means that the Swiss DBD donation rate remains relatively low in international comparisons [14, 15]. More importantly, it also means that organs donated for transplantation continue to be a scarce resource in Switzerland.

The aim of this study was twofold: First, to give an overview on key figures of the Swiss deceased organ donation and transplant activity between 2008 and 2017. Second, to compare the evolution of the donation efficiency of the Swiss donation program to that of the neighboring countries: Germany, Austria, Italy and France. Finally, we discuss the current situation of organ donation and transplantation in Switzerland in the context of future challenges.

## Methods

This study is an analysis of prospective registry data. It covers the period from 1 January 2008 to 31 December 2017 and includes all deceased organ donors in Switzerland. Patient data of all actual DBD donors and DCD (since 1 September 2011) donors were extracted from the Swiss Organ Allocation System (SOAS). Demographic data from the Federal Statistical Office was used for the calculation of the donation rate per million of population (pmp), based on the permanent resident population as of 1 January of each year (data available until 2016) [16].

The primary outcome of the study was the number of actual deceased donors (ADD) and basic donor characteristics per year. Per definition, an ADD is a consenting, eligible organ donor in whom an operative incision has been made with the intent of organ recovery for the purpose of transplantation [17]. The secondary outcome was the number of transplants enabled from these donors. The sub-analysis of the number of grafts transplanted per donor was limited to the following organ types: heart, lung, liver, kidney, and pancreas or pancreatic islets. In cases where more than one graft from one single organ was transplanted each graft was counted individually (this applies to left and right kidney, split livers, and to left and right lung lobes). Also included in the organ count were grafts from Swiss donors that were transplanted abroad within the framework of international organ exchange. Excluded from the organ count were transplants performed in Switzerland with grafts offered by foreign organ procurement organizations within the framework of international organ exchange, e.g. for super urgent listed patients awaiting a liver graft.

The international comparison of deceased donation program efficiency is based on the “donor conversion index” (DCI) methodology and data [14]. The DCI was developed in the framework of the Council of Europe’s European Committee on Organ Transplantation (CD-P-TO) by an international working group led by Swisstransplant. The DCI indicates how many ADD resulted from 100 deaths from a selection of causes associated with brain death, such as cerebrovascular accidents, anoxic brain damage and trauma resulting from traffic accidents [14, 17]. Mortality data was only available until 2015; therefore, 2016 and 2017 DCI values were calculated based on 2015 mortality data. The DCI is interpreted as follows: a DCI of 4% equals four ADD that resulted from 100 fatalities from the selected causes of death [14]. Some of the potential included in the DCI calculation may consist of patients who would not qualify for organ donation. For example, patients who are not dying in intensive care units but in palliative care units or patients in whom brain death diagnosis cannot be performed within the required timeframe. However, this is a general limitation of the DCI that equally applies to all countries included in our study.

Donor data for the year 2017 were retrieved from the websites of Eurotransplant (Austria, Germany) and Centro Nazionale Trapianti (Italy) [18, 19]. These figures represent utilized donors (deceased donors from whom at least one organ was transplanted [17]), as no ADD data was available. No donation data for 2017 was published by the French Agence de la biomédecine at the time of the writing of this article.

## Results

During the 10-year study period, there were 1116 ADD. The total number of ADD per year increased from 91 in 2008 to 145 in 2017 (+ 59%). While there was an overall increase, inter-annual donation figures showed some variance during the study period (Table 1). The reintroduction of the DCD program in late 2011 resulted in a growing percentage of DCD donors, reaching its maximum annual proportion (27%, *n* = 39) of ADD (*n* = 145) in 2017.

**Table 1:** Patient characteristics of deceased organ donors in Switzerland, 2008–2017

	2008	2009	2010	2011	2012	2013	2014	2015	2016	2017	2008–2017
Total ADD (n,%)	91 (100%)	104 (100%)	97 (100%)	102 (100%)	96 (100%)	110 (100%)	117 (100%)	143 (100%)	111 (100%)	145 (100%)	1116 (100%)
DBD	91 (100%)	104 (100%)	97 (100%)	99 (97%)	89 (93%)	98 (89%)	99 (85%)	127 (89%)	96 (86%)	106 (73%)	1006 (90%)
DCD	*n.a. (no DCD 2008–2010)*			3 (3%)	7 (7%)	12 (11%)	18 (15%)	16 (11%)	15 (14%)	39 (27%)	110 (10%)
Sex (n,%) and age											
Female	40 (44%)	47 (45%)	40 (41%)	49 (48%)	41 (43%)	37 (34%)	44 (38%)	68 (48%)	51 (46%)	56 (39%)	473 (42%)
Male	51 (56%)	57 (55%)	57 (59%)	53 (52%)	55 (57%)	73 (66%)	73 (62%)	75 (52%)	60 (54%)	89 (61%)	643 (58%)
Age (years, mean ± 1 SD)	52 ± 17.7	52 ± 17.3	51 ± 17.6	53 ± 19.6	54 ± 18.8	52 ± 19.2	51 ± 18.8	56 ± 17.5	52 ± 18.0	55 ± 17.3	53 ± 18.2

*Abbreviations*: *ADD* actual deceased donors, *DBD* donation after brain death, *DCD* donation after cardiocirculatory death, *n.a*., not applicable

Patient characteristics of ADD in Switzerland between 2008 and 2017 are given in Table 1. The proportion of female and male donors showed some inter-annual variances. Overall, 42% of donors were females (*n* = 473) and 58% males (*n* = 643). The mean donor age showed only minor variance during the 10 years analyzed, with an overall mean donor age of 53 ± 18.2 years.

### Donation and transplant activity

Table 2 shows annual donation and transplant activity data. The donation rate (number of ADD per million of population and year) was 12.0 pmp at the beginning of the study period and reached 17.4 pmp at the end (14.0 pmp overall mean donation rate). The average DBD donation activity was 12.6 pmp. It was highest in 2015 (15.4 pmp) and lowest in 2012 (11.2 pmp). The mean DCD donation rate was 1.9 pmp (2011–2017), with the lowest activity during the last four months of 2011 (0.4 pmp) and the highest in 2017 (4.7 pmp).

**Table 2:** Donation activity and transplants per donor and by organ type, 2008–2017

	2008	2009	2010	2011	2012	2013	2014	2015	2016	2017	2008–2017
Total ADD donation rate (pmp)^a^	12.0	13.5	12.5	13.0	12.1	13.7	14.4	17.4	13.3	17.4	14.0
DBD donation rate (pmp)^a^	12.0	13.5	12.5	12.6	11.2	12.2	12.2	15.4	11.5	12.7	12.6
DCD donation rate (pmp)^a^	*n.a. (no DCD 2008–2010)*			0.4	0.9	1.5	2.2	1.9	1.8	4.7	1.9^b^
Grafts transplanted per donor (ADD; mean number ± 1 SD)^c^	3.8 ± 1.3	3.5 ± 1.2	4.0 ± 1.3	3.8 ± 1.3	3.6 ± 1.6	3.2 ± 1.7	3.3 ± 1.6	3.1 ± 1.6	3.3 ± 1.6	2.9 ± 1.4	3.4 ± 1.5
DBD	3.8 ± 1.3	3.5 ± 1.2	4.0 ± 1.3	3.9 ± 1.3	3.7 ± 1.6	3.3 ± 1.8	3.5 ± 1.6	3.2 ± 1.6	3.5 ± 1.6	3.3 ± 1.4	3.5 ± 1.5
DCD	*n.a. (no DCD 2008–2010)*			2.3 ± 0.5	2.4 ± 0.9	2.6 ± 1.1	2.3 ± 1.0	2.2 ± 1.1	2.2 ± 1.1	2.1 ± 1.0	2.2^b^ ± 1.1
Graft utilization rate (ADD; number and percentage transplanted)^c^											
Heart [% based on DBD only]	30 (33%)	32 (31%)	42 (43%)	35 (35%)	35 (39%)	30 (31%)	37 (37%)	38 (30%)	37 (39%)	36 (34%)	352 (35%)
Lung	39 (43%)	40 (38%)	49 (51%)	52 (51%)	44 (46%)	42 (38%)	49 (42%)	50 (35%)	49 (44%)	31 (21%)	445 (40%)
Liver	75 (82%)	81 (78%)	86 (89%)	89 (87%)	78 (81%)	86 (78%)	96 (82%)	117 (82%)	83 (75%)	112 (77%)	903 (81%)
Kidney [% based on 2 kidneys per donor]	165 (91%)	184 (88%)	177 (91%)	178 (87%)	150 (78%)	164 (75%)	177 (76%)	216 (76%)	177 (80%)	226 (78%)	1814 (81%)
Pancreas and pancreatic islets	26 (29%)	19 (18%)	29 (30%)	28 (27%)	30 (31%)	30 (27%)	24 (21%)	20 (14%)	22 (20%)	21 (14%)	249 (22%)

*Abbreviations*: *ADD* actual deceased donors, *pmp* per million of population, *DBD* donation after brain death, *DCD* donation after cardiocirculatory death, *n.a*., not applicable

^a^The 2017 donation rates are based on 2016 population (most recent data available from the Federal Statistical Office)

^b^Mean of years 2011–2017

^c^In cases where more than one graft from one single organ was transplanted each graft is counted individually (this applies to left and right kidney, split livers, and to left and right lung lobes). Includes organs from Swiss donors transplanted abroad within the framework of international organ exchange

The overall average number of transplants enabled per DBD donor was 3.5 ± 1.5, and 2.2 ± 1.1 grafts transplanted per DCD donor. The mean number of transplants per DBD donor showed some inter-annual variance, with slightly higher rates at the beginning of the evaluation period. In DCD donors, the transplant rate per donor increased until 2013 and showed a downward trend thereafter.

The total number of hearts, lungs, livers, kidneys, and pancreata or pancreatic islets transplanted during the study period was 3763 from all ADD. Figures not included in the table show that 94% (*n* = 3541) of transplants resulted from DBD donors, and 6% (*n* = 222) from DCD donors. Of all grafts transplanted (*n* = 3763), 48% were kidneys (*n* = 1814), 24% livers (*n* = 903), 12% lungs (*n* = 445), 9% hearts (*n* = 352), and 7% pancreata or pancreatic islets (*n* = 249). (The percentages per organ type shown in the table represent the graft utilization rate.).

Table 2 also shows that the graft utilization rate (number of grafts transplanted expressed as a percentage of the number of ADD) varied considerably between the organ types, as well as in some organ types over time. During the entire study period, the graft utilization rate was highest for kidney and liver (81% both), followed by lung (40%), heart (35%; DBD only), and pancreas or pancreatic islets (22%).

### Donation efficiency in comparison with neighboring countries

A comparison of different countries’ donation rates pmp has various limitations as discussed in detail in previous publications [14, 20–26]. The major shortcoming of the donation rate pmp is that it cannot give a proper account of the potential for deceased donation. The donation rate is calculated based on a country’s entire living population and not on the actual mortality relevant to organ donation. An evaluation of a donation program’s performance (i.e., its conversion efficiency of the potential into donors), however, needs to take place in the context of a realistic estimate of the potential for deceased donation. If the potential is not being accounted for, a comparison will be biased, as countries with a higher potential (mortality from relevant causes) are likely to reach higher donation rates pmp, even with a less efficient donation program. In this study, we used DCI methodology and data [14] as a metric for the assessment and comparison of donation efficiency.

**Fig. 1:**
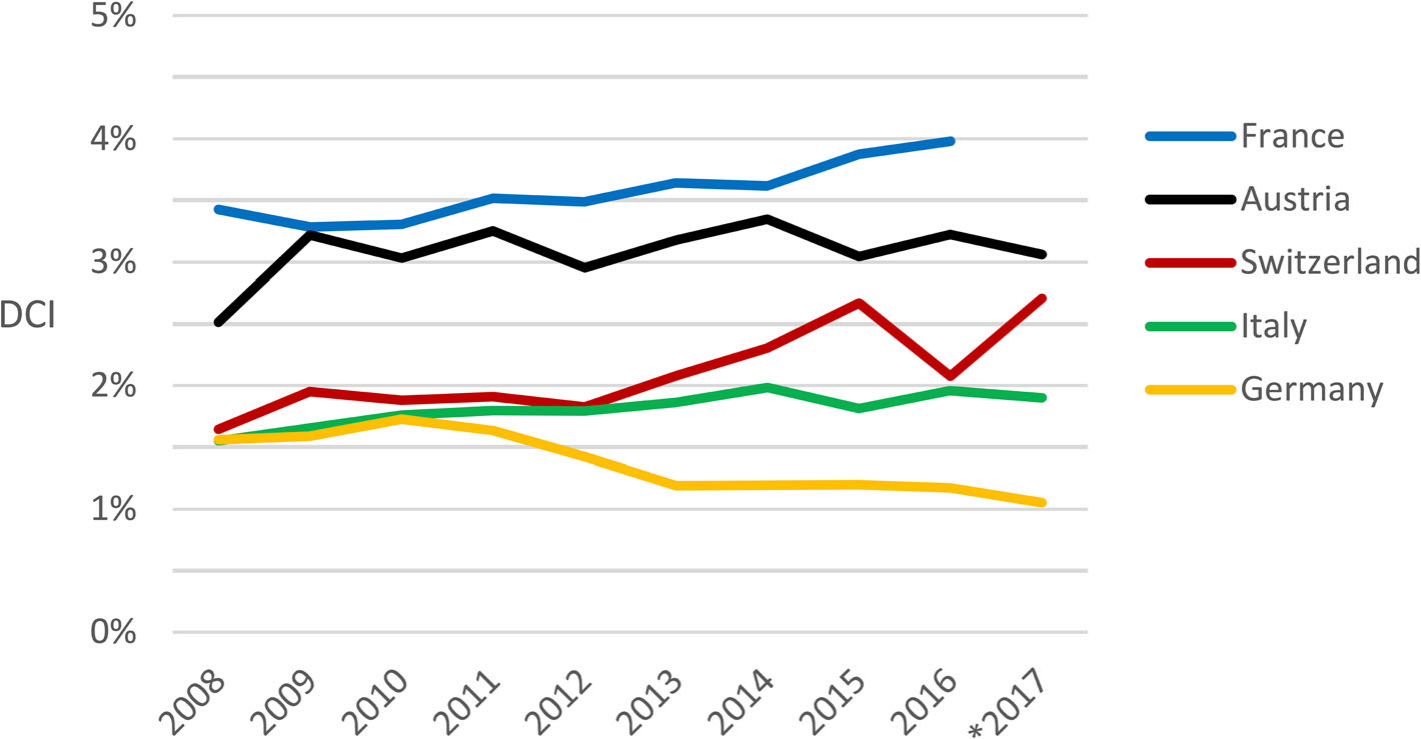
Donation efficiency evolution in Switzerland and neighboring countries, 2008–2017. * 2017 donation data for Austria, Italy and Germany are utilized donors. The actual DCI of these donation programs may likely be 0.1–0.2 percentage points higher. 2008–2015 data retrieved from [14]; sources of 2016–2017 data: [15, 18, 19, 71] and SOAS

Figure 1 shows the DCI evolution for Switzerland, Germany, Austria, Italy, and France between 2008 and 2017. The DCI indicates how many ADD resulted from 100 deaths from various causes associated with brain death, such as cerebrovascular accidents, anoxic brain damage, trauma resulting from traffic accidents. According to the DCI, France and Austria had more efficient donation programs than Switzerland in each year of the study period. As in all countries (except for Germany), the Swiss DCI increased during the last decade. At the end of the evaluation period, the efficiency of the Swiss donation program has increased by 69% compared with the beginning (2.7% DCI in 2017 vs. 1.6% DCI in 2008). Donation efficiency in Switzerland was relatively stable (and similar to the efficiency of the German and Italian donation programs) until 2011. Between 2012 and 2017, the Swiss DCI increased most notably, showing a growth of 50% (1.8% DCI in 2012 vs. 2.7% DCI in 2017). The efficiency of the Italian donation program also improved during the evaluation period; however, the increase was less pronounced. Germany was the only one among Switzerland’s neighboring countries that showed a negative DCI trend over the evaluation period.

## Discussion

Our analysis shows that both the deceased donation activity and efficiency of the Swiss deceased organ donation program increased substantially during the last decade. Despite the measures implemented so far, the efficiency of the Swiss donation program stays below the French and Austrian programs. As a result of the improved efficiency in the conversion of the potential into donors, the Swiss program showed a moderately higher performance than the Italian program at the end of the study period.

### Donation and transplant activity

The Swiss deceased donation activity (annual donation rate; range 12.0–17.4 pmp, mean 14.0 pmp) during the study period was lower than in Italy, France and Austria which had annual donation rates > 20 pmp (Italy, Austria), and > 23 pmp (France) in each year between 2008 and 2015 [14].

Patient characteristics of Swiss donors during the decade preceding our study period showed a marked increase in the mean age of donors [27]. In our study period, the mean donor age remained roughly stable (similar to the most recent historic data). Also, the mean donor age of Swiss donors between 2008 and 2017 is comparable to the mean age of French donors in recent years [28]. In 2016 (latest ADD data published for the neighboring countries), the average number of transplants enabled per donor in Switzerland (3.3) was similar to Germany (3.4) and Austria (3.1), and higher than in France (2.7) and Italy (2.3) [15]. This means that in countries with relatively low donation rates, a higher transplant rate per donor may compensate, at least partially, for the low donation activity. One should keep in mind, however, that a more liberal use of extended criteria donors (i.e., older donors and/or donors whose medical conditions are considered suboptimal) may be only suitable for selected subgroups of recipients, and may impact the transplant outcome [29–32]. When comparing average numbers of transplants enabled per donor, it is also important to consider the fact that the percentage of DCD donors in Switzerland (14% in 2016) was markedly higher than in the other countries with a DCD program (Italy, 1.4%; Austria, 2.8%; France, 4.8%) [15]. Because in the countries included in the study, the heart may be transplanted only from DBD donors, a larger proportion of DCD donors leads per se to a reduced average number of transplants enabled in the totality of DBD and DCD donors.

As DCD may be considered as supplementary to DBD, the reintroduction of the Swiss DCD program has successfully contributed to the increase of the number of organs available for transplantation [3, 33]. As a matter of fact, 222 grafts from DCD donors have been transplanted to patients on the Swiss waiting list since September 2011. This represents additional life-years and quality of life gained by a considerable number of recipients. Because kidney transplants are cost-saving compared with dialysis for patients with end-stage renal failure, additional kidneys from DCD donors have also a beneficial effect on health care expenditure [34]. Long-term outcomes of transplants with kidneys and lungs from DCD donors seem to be similar compared with grafts from DBD donors [35–42]. Liver transplant outcomes from DCD donors have been reported as sometimes inferior compared to DBD, especially after prolonged warm ischemia time [35, 43–45].

### Donation efficiency in comparison with neighboring countries

The improved efficiency of the Swiss donation program suggests that the measures implemented were effective. A considerable proportion of the DCI increase, however, was due to the reintroduction of DCD. The DBD efficiency also increased over time, but less pronouncedly. Due to the relatively small absolute number of donors per year in small countries, annual DCI variations may occur (such as in 2016 in Switzerland or the see-sawing of the Austrian DCI). Yet, the evolution of the Swiss DCI shows a clear trend of increasing donation efficiency since 2012.

When considering possible reasons for differences in DCI between the countries, the consent rate (proportion of patients and/or families consenting to organ donation) is one factor that has a major impact on the DCI. Even though there is no internationally standardized way to report consent rates, published data show important differences between countries [5–8, 46, 47]. According to 2012–2016 SwissPOD data, the overall consent rate of patients who deceased in the participating Swiss intensive care units was 43.5% [7]. This suggests that roughly half of the donor potential was lost due to either patients or their next of kin not consenting to organ donation. In contrast, recent data from France, Austria and Italy (no data available for Germany) indicate that in these countries, only approximately one third of the potential was not converted because of patients or next of kin not consenting to organ donation [15, 28]. If the consent rate in Switzerland was similar to the approximately 70% consent rate in France, Austria and Italy, the 2017 Swiss DCI would be estimated at about 3.8% (2.7% divided by 0.5 and multiplied by 0.7). Therefore, improving the consent rate in Switzerland should result in a significant additional efficiency increase of the Swiss donation program.

### Evaluation of the current situation and outlook

In comparison with the neighboring countries and as discussed above, the performance of the Swiss organ donation and transplant program can be considered equal or slightly better, as the average number of transplants enabled per donor is similar to figures in Germany and Austria, and higher than in France and Italy. The relatively high number of transplants per donor contributes substantially to the overall performance of the Swiss donation program as it ensures an optimized use of the limited donor pool.

One factor that clearly has a negative impact on the donation activity and efficiency in Switzerland is the low consent rate which is in contrast to the largely positive attitude towards organ donation and transplantation among the Swiss population [48–51]. Under the assumption that the consent policy (explicit consent in Switzerland and Germany, presumed consent in France, Austria, and Italy) may have an impact on the consent rate, it seems appropriate to consider changing the Swiss policy in order to achieve the Federal action plan’s goal of a refusal rate below 40% [10, 52]. The fact that the consent rate in Switzerland has been roughly stable during the study period indicates, however, that the growth in efficiency may have resulted, at least to some degree, from the measures implemented. Naturally, there is no guarantee that a change in the system to presumed consent in Switzerland would per se lead to an increased rate of donors. Previous studies have pointed out that awareness of the consent policy may play an important role, and that changing the policy to presumed consent requires adequate and continuous information of the population [53–57]. Efforts in Switzerland during the last decade have led to an efficient organ donation process, and the consent rate remains the action area with the most potential for a growth in the numbers of donors.

Action areas of the action plan included the training of healthcare professionals, the implementation of standardized processes and quality management, as well as optimizing structures and allocating resources in the hospitals [12, 58]. Swisstransplant and the CNDO were commissioned by the FOPH and the cantons to accomplish these tasks.

The training of health care professionals has been standardized and integrated in a newly created blended learning program, established in 2015. The blended learning program consists of ten e-learning modules, and two face-to-face courses (“communication with next of kin” and “medicine and quality in the donation process”). Since its introduction, roughly one thousand health care professionals have been enrolled in the program. Hospital staff in charge with tasks related to organ donation (the 150 local organ and tissue donation coordinators) are required to complete the program and pass the final exam within two years.

As an additional measure and since mid-2016, earmarked funds are being allocated to the local organ and tissue donation coordinators. The allocation of earmarked funds has been a crucial step in the optimization of the donation process. First, because these funds recompense defined percentages of coordinators’ working time. Second and related, it is bound to enhanced accountability concerning the specific tasks of the coordinators. All hospitals with an accredited intensive care unit are contractually obliged to designate a local organ and tissue donation coordinator. To ensure a 24/7 donation coordination, the hospitals are clustered into donation networks, with each network having an on-call service for donation coordination.

The optimization of structures and processes included, among others, the involvement of accident and emergency departments as well as paramedics, and the ongoing training of staff. While the action plan mainly focused on optimizing the prerequisites for donation, one should bear in mind that there are other areas where there may be additional potential for improvement. For example, in donor management, procurement and ex-vivo conditioning [59–62], or in the prevention and treatment of diseases leading to terminal organ failure which could result in less patients who need to be waitlisted for an organ transplantation [63–65].

Finally, an evaluation of the performance of the Swiss organ donation and transplant program should take into account that its ultimate goal is to enable a maximum of transplants with a successful outcome. Outcome data for transplant recipients in Switzerland are generally similar or slightly better than the results reported in large international registries [66–69]. In view of the high average number of organs transplanted per donor, and the relatively high mean donor age (e.g., compared with US data [70]), this is an excellent result in terms of quality of care provided by the Swiss transplant centers.

### Study strengths and limitations

Our study has several strengths and limitations. We consider it the main strength that it provides key figures on the evolution of the Swiss organ donation and transplant program during the last decade. These data, in combination with the assessment of the donation efficiency, should allow the reader to gain a general overview on the performance of the Swiss organ donation program. It also provides the context for a comparison with the performance of the donation programs in the neighboring countries.

Limitations of the DCI include that the mortality from the selected causes allows only for an approximation of the potential, and that international mortality data was only available until 2015 (for a detailed discussion of the DCI’s limitations see [14]). The 2016 and 2017 DCI values of all countries included in our study are based on the latest mortality data available (2015). The 2017 DCI values of Austria, Italy, and Germany were calculated based on utilized donors instead of ADD, as at the time of the writing of this paper and to the best of our knowledge, ADD data had not yet been published. This may lead to a slightly underestimated DCI for these donation programs in 2017. A final word of caution should be given regarding the international comparability of consent rates. Due to non-standardized modalities of consent rate reporting, the data may not be completely comparable.

## Conclusions

Even though the Swiss donation activity and efficiency have been substantially improved during the last decade, it remains below the French and Austrian donation rates and DCI. This may be explained, at least in part, to the considerably lower consent rate in Switzerland. The increased donation efficiency in Switzerland suggests that the measures implemented so far (allocation of earmarked funds to local donation coordinators, optimized structures, training of healthcare professionals) have been effective. In view of the fact that the consent rate has been roughly stable at a low level, the Swiss intensive care units were able to increase the performance considerably in terms of donor detection and referral. The most important factor that negatively impacts the donation activity and efficiency is the high percentage of family refusals. This issue needs to be addressed in order to achieve the goal of more organs available for transplantation.

## Abbreviations

ADD: Actual deceased donors
CNDO: Comité National du Don d’Organes
DBD: Donation after brain death
DCD: Donation after cardiocirculatory death
DCI: Donor conversion index
FOPH: Federal Office of Public Health
pmp: per million of population
SOAS: Swiss Organ Allocation System
SwissPOD: Swiss Monitoring of Potential Donors
